# Lung and Heart Diseases Are Better Predicted by Pack-Years than by Smoking Status or Duration of Smoking Cessation in HIV Patients

**DOI:** 10.1371/journal.pone.0143700

**Published:** 2015-12-09

**Authors:** Giovanni Guaraldi, Paolo Raggi, André Gomes, Stefano Zona, Enrico Marchi, Antonella Santoro, Giulia Besutti, Riccardo Scaglioni, Guido Ligabue, Jonathon Leipsic, Paul Man, Don Sin

**Affiliations:** 1 Modena HIV Metabolic Clinic, UNIMORE, Modena, Italy; 2 Mazankowski Alberta Heart Institute, University of Alberta, Edmonton, Canada; 3 Infectious Diseases Department, Hospital Garcia de Orta, Almada, Portugal; 4 Radiology Unit, UNIMORE, Modena, Italy; 5 Department of Radiology, University of British Columbia, Vancouver, Canada; 6 Department of Medicine (Respiratory Division), University of British Columbia, Vancouver, Canada; University of Missouri-Kansas City, UNITED STATES

## Abstract

**Background:**

The objective of this study was to assess the relationship of pack-years smoking and time since smoking cessation with risk of lung and heart disease.

**Methods:**

We investigated the history of lung and heart disease in 903 HIV-infected patients who had undergone thoracic computed tomography (CT) imaging stratified by smoking history. Multimorbidity lung and heart disease (MLHD) was defined as the presence of ≥ 2 clinical or subclinical lung abnormalities and at least one heart abnormality.

**Results:**

Among 903 patients, 23.7% had never smoked, 28.7% were former smokers and 47.6% were current smokers. Spirometry indicated chronic obstructive pulmonary disease in 11.4% of patients and MLHD was present in 53.6%. Age, male sex, greater pack-years smoking history and smoking cessation less than 5 years earlier vs. more than 10 years earlier (OR 2.59, 95% CI 1.27–5.29, p = 0.009) were independently associated with CT detected subclinical lung and heart disease. Pack-years smoking history was more strongly associated with MLHD than smoking status (p<0.001).

**Conclusions:**

MLHD is common even among HIV-infected patients who never smoked and pack- years smoking history is a stronger predictor than current smoking status of MLHD. A detailed pack-years smoking history should be routinely obtained and smoking cessation strategies implemented.

## Introduction

Smoking and HIV infection are independently associated with cardiovascular and respiratory diseases [1]. Regrettably, the rates of smoking are twofold higher among HIV-infected patients compared to the general population (42% versus 21%, respectively) and HIV-infected patients are also less likely to quit smoking (quit ratio, 32.4% vs. 51.7%)[2, 3]. In the Western world, cigarette smoking is the single most important modifiable risk factor for preventing lung cancer, chronic obstructive pulmonary disease (COPD) and coronary heart disease[4, 5]. Among HIV-infected persons, health hazards of smoking may be greater. For instance, the relative risk of lung cancer is 3.5 times higher in HIV-infected patients compared with non-infected individuals,[6], the prevalence of COPD is increased by approximately 7–21%[7, 8] and cardiovascular disease (CVD) by 76%[9]. Although mechanisms by which HIV infection enhances the risk of these conditions are in general unknown, anti-retroviral therapy (ART), immune hyper-activation and premature senescence have been implicated in the pathogenesis of heart and lung disease [10].

Despite the importance of smoking in the pathogenesis of lung cancer, COPD, and coronary artery disease, the impact of cumulative cigarette exposure on the risk of these conditions among HIV-infected persons has not been well investigated [11]. To date, most published studies of HIV-infected persons have focused largely on current smoking status, with no or little attention to cumulative (lifetime) exposure. The primary objective of this study was to determine the relationship of lifetime smoking history as a measure of cumulative tobacco exposure and time since smoking cessation with risk of lung and heart disease in a large group of HIV-infected individuals.

## Methods

### Study population

This was an observational cross-sectional study of 903 consecutive HIV-infected patients followed at the University of Modena HIV metabolic clinic (MHMC) between January 2006 and September 2012. The details of this cohort have been previously published[12]. In brief, MHMC is a multidisciplinary centre for the diagnosis and treatment of non-infectious co-morbidities in HIV-infected patients. Patients are assessed (free of charge) for lipodystrophy, diabetes mellitus, hypertension, cardiovascular disease (CVD), osteoporosis, kidney failure, and liver and lung disease through comprehensive clinical evaluation and a select number of diagnostic interventions including dual energy X-ray absorptiometry (DEXA), chest and abdominal computed tomography (CT) scans and biochemical blood tests. In 2011 pulmonary function testing was added as described elsewhere [13]. The current project received approval from the provincial institutional ethics review board at the University of Modena (Comitato Etico Provinciale) and all subjects provided a written consent to participate in the study.

In the present analysis, we included patients who were older than 18 years, had serologically documented HIV-1 infection, and had been treated for at least 12 months with ART prior to enrolment. Exclusion criteria were active lung or heart disease at the time of the enrolment and pregnancy.

### Definition of Clinical Heart and Lung Disease

Clinical lung diseases included COPD and lung cancer diagnosed at the time of patient visit. We defined COPD using a ratio of post-bronchodilator forced expiratory volume in one second to forced vital capacity (FEV_1_/FVC) of less than 0.70. Pulmonary function tests were performed using a Jaeger Flow Screen spirometer and Jaeger Master Screen plethysmography (RA.Med srl Medical technology) according to the ATS/ERS recommendations[14], Since spirometry was introduced only in 2011, lung function parameters were available in 39% of the cohort.

Lung cancer was defined based on standard histology. In this cohort, lung cancer was detected through the use of screening CT scans.

Clinical heart disease was defined as myocardial infarction (MI), which was diagnosed based on MONICA criteria, [15] by patient history or by detection of an area of myocardial scarring on cardiac CT scans at the time of the patient visit.

### Definition of Subclinical Heart and Lung Disease

Subclinical lung and heart conditions were detected with CT imaging. For this purpose, a cranio-caudal sequence of 30–40 consecutive axial slices of the heart was acquired with a 64-slice volume CT multidetector scanner (GE Medical Systems, Milwaukee, WI). All images were obtained during a single breath hold using 320 mAs and 140 Kv. Image acquisition was prospectively triggered at 80% of the R-R interval on the surface electrocardiogram. A section thickness of 2.5 mm, a field of view of 20 cm^2^, and a matrix of 512 x 512 were used to reconstruct the raw image data, yielding a nominal pixel size of 0.39 mm^2^ and a voxel of 0.4 mm^3^.

CT Images were evaluated for findings of pulmonary emphysema, and bronchiolitis as defined by the Fleischner Society glossary of terms by 3 radiologists by consensus reading using an offline CT workstation (AW 4.4, GE Healthcare, Milwaukee, WI). [16]. Subclinical lung conditions were detected based on semi-quantitative scores for emphysema (i), bronchiolitis (ii), bronchial wall thickening (iii), bronchiectasis (iv) and non-calcified lung nodules (v)[17].

Subclinical coronary vascular abnormality was assessed with coronary artery calcium (CAC). CAC was quantified according to the Agatston technique [18]. A CAC score cut off >100 was considered evidence of significant coronary artery disease risk, as this threshold has been shown to correlate closely with adverse cardiac events in the general population.

### Multimorbidity lung and heart disease

Multimorbidity lung and heart disease (MLHD) was defined by the presence of ≥ 2 clinical or subclinical lung abnormalities and at least one cardiovascular condition (either CAC >100 or previous myocardial infarction). This composite end-point was used to capture the impact of cigarette smoking on the complex interaction of heart and lung diseases (both clinical and subclinical).

### Smoking history

Smoking information was collected for all patients, including current smoking status, duration, intensity of smoking during different life periods, time since cessation and age at smoking initiation. The pack-years of smoking were calculated by multiplying the average number of packs of cigarettes smoked per day (intensity) by the number of years the person had smoked. Pack-years smoking history estimates the lifetime exposure to tobacco. Smoking status was classified into 3 groups: never smoked, former smoker and current smoker.

To determine the impact of smoking, the study cohort was subdivided into 7 groups: never smokers (NS), former smokers with a pack-years history ≤10 (FS<10PY), former smokers with a pack-years history of 11–20 (FS:11–20PY), former smokers with a pack-years history >20 (FS>20PY), current smokers with a pack-years history ≤10 (CS<10PY), current smokers with a pack-years history of 11–20 (CS:11–20PY), and current smokers with a pack-years history >20 (CS>20PY). In the former smoker category, time since smoking cessation was categorized into 3 groups: <5 years, 5–10 years, >10 years of quitting. Information regarding use of illicit intravenous drugs was also collected.

### Covariates

Demographic and clinical data at the time of CT scanning were captured. HIV related variables were collected by patient medical chart review and included: nadir and current CD4+ T lymphocyte cell count/mm^3^ (CD4), time since HIV diagnosis and cumulative exposure to antiretroviral drugs: protease inhibitors (PIs), nucleoside reverse transcriptase inhibitors (NRTIs) and non-nucleoside reverse transcriptase inhibitors (NNRTIs). Efficacy of ART was determined by measuring plasma HIV-1 RNA at the time of CT scan and dichotomized as below or above the limit of detection (<40 c/mL). Previous AIDS diagnosis was defined according to the Centers for Disease Control group “C” category[19]. Anthropometric measurements were: waist circumference, body mass index (BMI, calculated as weight in kilograms divided by the square of height in meters). Lipodystrophy was defined according to the HOPS categories [20]. Metabolic variables were collected following an overnight fast. The measured serum lipids included total cholesterol, low-density lipoprotein cholesterol, high-density lipoprotein cholesterol, and triglycerides. Insulin resistance was calculated using the homeostasis model assessment equation: HOMA-IR 1/4 [fasting insulin (mU/ml) x fasting glucose (mmol/l)/22.5]. The Framingham risk score (FRS) was calculated for each patient using equations developed by the Adult Treatment Panel III[21].

### Statistical Analyses

Patient characteristics were compared among groups using ANOVA and Kruskal-Wallis test for continuous normally distributed and non-normally distributed variables, respectively. Fisher’s exact test was used to compare categorical variables. The prevalence of clinical and subclinical manifestations was compared among groups using a Fisher’s exact test: post-hoc analyses were performed using Bonferroni adjustments. Trends in prevalence of pulmonary and cardiovascular diseases were tested using Cochran–Armitage test. Factors associated with MHLD were assessed using univariate and multivariable logistic regression analyses. Covariates included in the final model were: age, sex, HOMA-IR, HIV transmission route, and smoking history. Receiver operating characteristics (ROC) analyses were used to compare the association between disease states (lung, heart, and MLHD) and current smoking status or pack-years of smoking. C-statistics were used to compare the ROC curves of various models. Statistical significance level was set at p<0.05 (two-tailed). All statistical analyses were performed using STATA 13.1 for Mac (StataCorp, College Station, TX, US).

## Results

### Participants

Of the 903 participants, 263 (29%) were women and 99% were Caucasians. The mean patient age (SD) was 48 years (SD 7.5). Anthropometric, laboratory values and lifestyle characteristics are described in Table 1. There were 214 patients (23.7%) who had never smoked, 259 (28.7%) were former smokers and 430 (47.6%) were current smokers. Pack-years of smoking were greater in current than in former smokers (median 22.5 pack-years versus 14.0 pack-years; p<0.001). Using our smoking categories 214 (23.7%) patients were NS, 102 (11.3%) FS<10PY, 63 (7.0%) FS:11–20PY, 94 (10.4%) FS>20PY, 60 (6.6%) CS<10PY, 120 (13.3%) CS:11–20PY, and 250 (27.7%) CS>20PY.

**Table 1:** Demographic and clinical characteristics of the study group by smoking status.

	Never smoker (n = 214)	Former smoker (n = 259)	Current smoker (n = 430)	p
Women, n (%)	59 (27.6%)	79 (30.50)	125 (29.07)	0.789
Age, mean (sd)	48.44 (8.72) *	50.16 (7.41) ^	47.92 (6.50) *^	<0.001
Sedentary life, n (%)	103 (49.28) *	128 (50.59) ^	246 (58.99) *^	0.002
Alcohol <20 g/day, n (%)	60 (28.71) *^	103 (40.71)^	188 (45.08) *	<0.001
Waist circumference, mean, cm (sd)	88.88 (10.48) *	88.37 (9.50) *^	85.84 (9.15) *^	<0.001
BMI, mean (sd)	24.52 (3.87) *	24.07 (3.29) *^	23.15 (3.77) ^	<0.001
Lipodystrophy				0.013
Lipoatrophy, n (%)	52 (33.77) *	70 (40.94)	138 (47.59) *	
Lipohypertrophy, n (%)	17 (11.04)	18 (10.53)	18 (6.21)	
Mixed form, n (%)	55 (35.71) *	61 (35.67)	76 (26.21) *	
Fasting glucose, mg/dL, mean (sd)	97.41 (20.30)	100.94 (25.90) *	94.82 (23.65) *	0.005
HOMA-IR, median (IQR)	2.24 (1.46–3.78) *	2.61 (1.68–4.43) ^	1.81 (1.17–3.12) *^	<0.001
Triglycerides, mg/dL, median (IQR)	148.5 (105–220)	147 (98–209)	147 (106.5–213.5)	0.789
Total Cholesterol, mg/dL, mean (sd)	199.74 (47.24)	198.20 (47.00)	194.50 (41.21)	0.327
HDL-C, mg/dL, mean (sd)	47.90 (15.35)	49.20 (15.25) ^	45.29 (13.73) ^	0.002
LDL-C, mg/dL, mean (sd)	120.17 (36.83)	117.90 (35.64)	117.51 (34.30)	0.671
CD4 Nadir, cells/microL, median (IQR)	188.5 (76–292) *	141 (45–250) *^	187.5 (82–280) ^	0.002
Current CD4, cells/microL median (IQR)	575 (437–713)	527 (413–703) *	594 (441–824) *	0.037
Cumulative exposure to NRTIs, months, median (IQR)	118 (68–153) *	138 (94–181) *	131 (69–171)	0.030
Cumulative exposure to PIs, months, median (IQR)	50 (8–81)	51 (22–91)	59 (19–99)	0.556
Cumulative exposure to NNRTIs, months, median (IQR)	28 (3–66)	23 (0–71)	23 (0.61)	0.602
HTN, n (%)	86 (40.19)	121 (46.72) ^	147 (34.19) ^	0.005
T2DM, n (%)	32 (14.95)	48 (18.53) ^	44 (10.23) ^	0.008
FRS, median (IQR)	3 (1–7) *	4 (1–7) ^	7 (3–12) *^	<0.001
Age of smoking initiation		16 (15–18)	16 (15–18)	0.921
Pack-Years, median (IQR)	0 (0)	14 (7–26.25)	22.5 (14.6–30.6)	<0.001
0–10 PY, n (%)		102 (39.38)	60 (13.95)	
11–20 PY, n (%)		63 (24.32)	120 (27.91)	
>20 PY, n (%)		94 (36.29)	250 (58.14)	

BMI- body mass index; FRS- Framingham Risk Score; HDL-C- high-density lipoprotein cholesterol; HOMA-IR- homeostasis model assessment of insulin resistance; HTN- hypertension; IQR- interquartile range; LDL-C- low-density lipoprotein cholesterol; NNRTIs- non-nucleoside reverse-transcriptase inhibitors; NRTIs- nucleoside reverse transcriptase inhibitors; PIs- protease inhibitors; PY- pack-years; sd- standard deviation; T2DM- type 2 diabetes mellitus. Symbols (* and ^) represent statistical significant differences between groups in the same line. If two cells in the same line have the same symbol means that that comparison is statistically significant.

### Respiratory diseases

**Fig 1:**
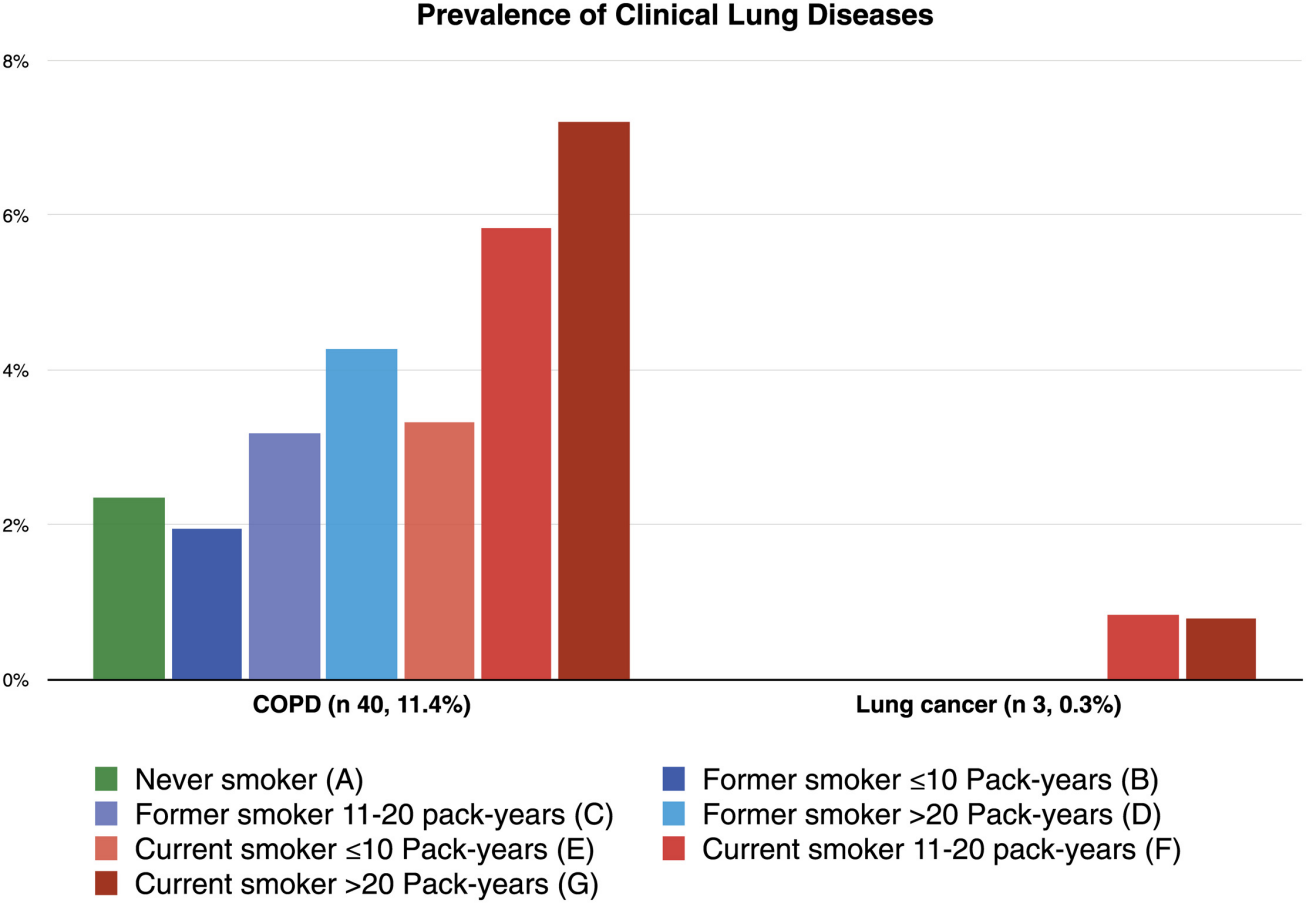
Prevalence of clinical lung diseases by smoking status and pack-years groups. The letters represent each group and when placed on the top of a bar it represents a statistical difference between that bar and the group the letter represent.

**Fig 2:**
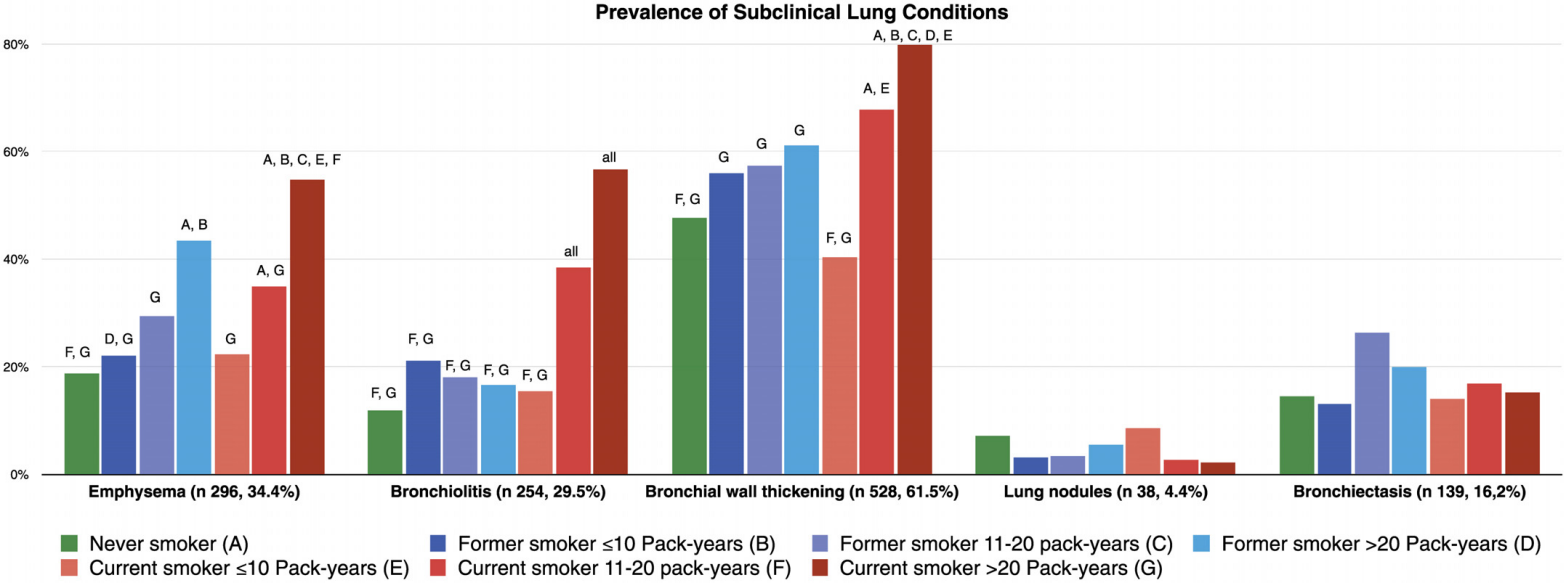
Prevalence of subclinical lung conditions by smoking status and pack-years groups. The letters represent each group and when placed on the top of a bar it represents a statistical difference between that bar and the group the letter represent.

**Fig 3:**
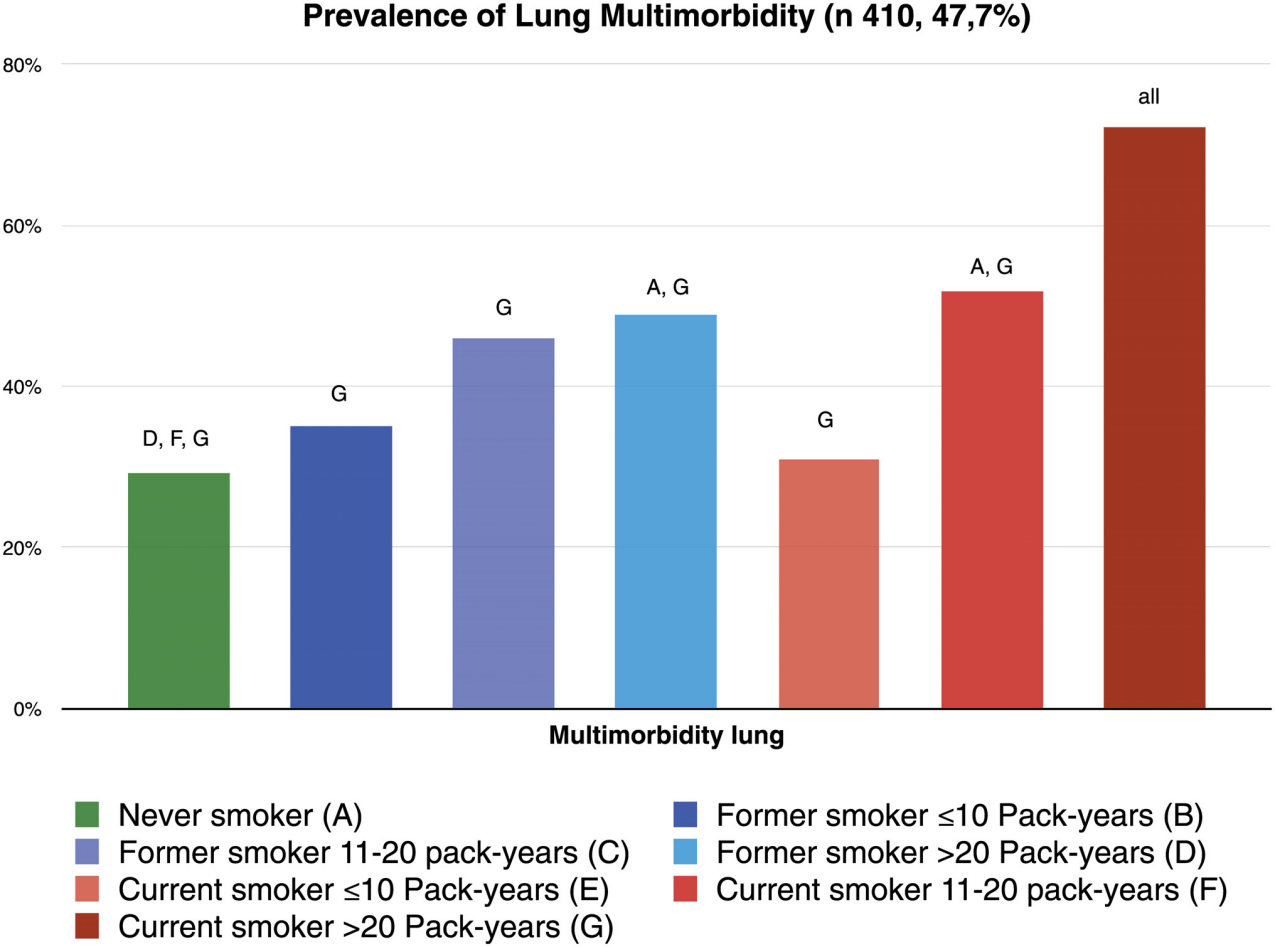
Prevalence of multimorbidity lung diseases by smoking status and pack-years groups. The letters represent each group and when placed on the top of a bar it represents a statistical difference between that bar and the group the letter represent.

Three lung nodules (0.3%) suspicious of carcinoma were found on CT imaging. Histological examination confirmed the diagnosis of lung cancer in all 3 patients. Of the 350 patients who underwent pulmonary function testing, 40 (11.4%) were diagnosed with COPD. Among these patients, 28 had emphysema on CT imaging. Among the 12 patients without emphysema, 10 had CT evidence of bronchial wall thickening. Among the 860 patients without clinical COPD and/or lung cancer, CT scanning identified the following pulmonary conditions: 296 patients (34.4%) had emphysema, 254 (29.5%) had bronchiolitis, 38 (4.4%) had non-calcified lung nodules, 528 (61.4%) had significant bronchial wall thickening and 139 (16.2%) had bronchiectasis. Multimorbidity lung disease (MLD) was found in 410 (47.7%) patients. The distribution of clinical, subclinical and multimorbidity lung disease by smoking status and pack-year groups is shown in Figs 1, 2 and 3 respectively. There was a trend towards a higher risk of clinical, subclinical and multimorbidity lung disease with increasing pack-years of smoking. The highest prevalence was found in current smokers.

### Cardiovascular disease

**Fig 4:**
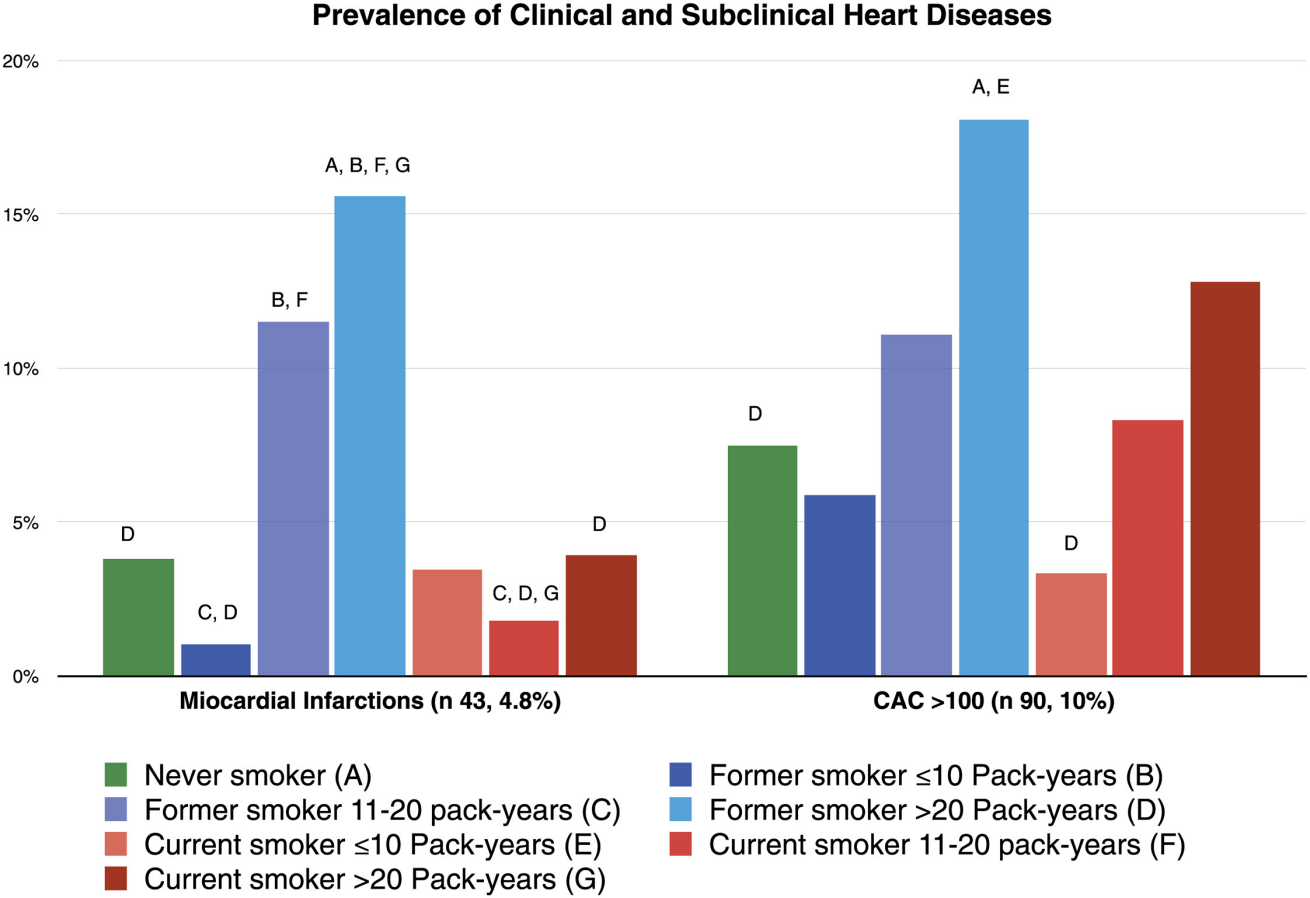
Prevalence of clinical and subclinical heart diseases by smoking and pack-years groups.

Forty-three patients (4.7%) had evidence of prior myocardial infarction (MI): 30 (3.3%) had a clinical history of MI and 13 (1.4%) demonstrated areas of myocardial scarring on CT imaging. By smoking status, 8 MIs occurred in never smokers, 22 in former smokers and 13 in current smokers. Of note 8 patients among the former smokers were active smokers at the date of the myocardial event. CAC>100 was found in 90 (10%) patients; 16 (7.5%) among never smokers, 30 (11.5%) among former smokers and 44 (10.2%) among current smokers. One-hundred twenty-three (13.6%) patients had CAC>100 and/or previous MI. Distribution of MIs by smoking status and pack-years category is shown in Fig 4. There was a trend toward a higher prevalence of clinical and subclinical heart disease with increasing pack-years of smoking, both in former (p<0.001) and in current smokers (p<0.001). The highest prevalence was found in current smokers.

### MLHD

**Fig 5:**
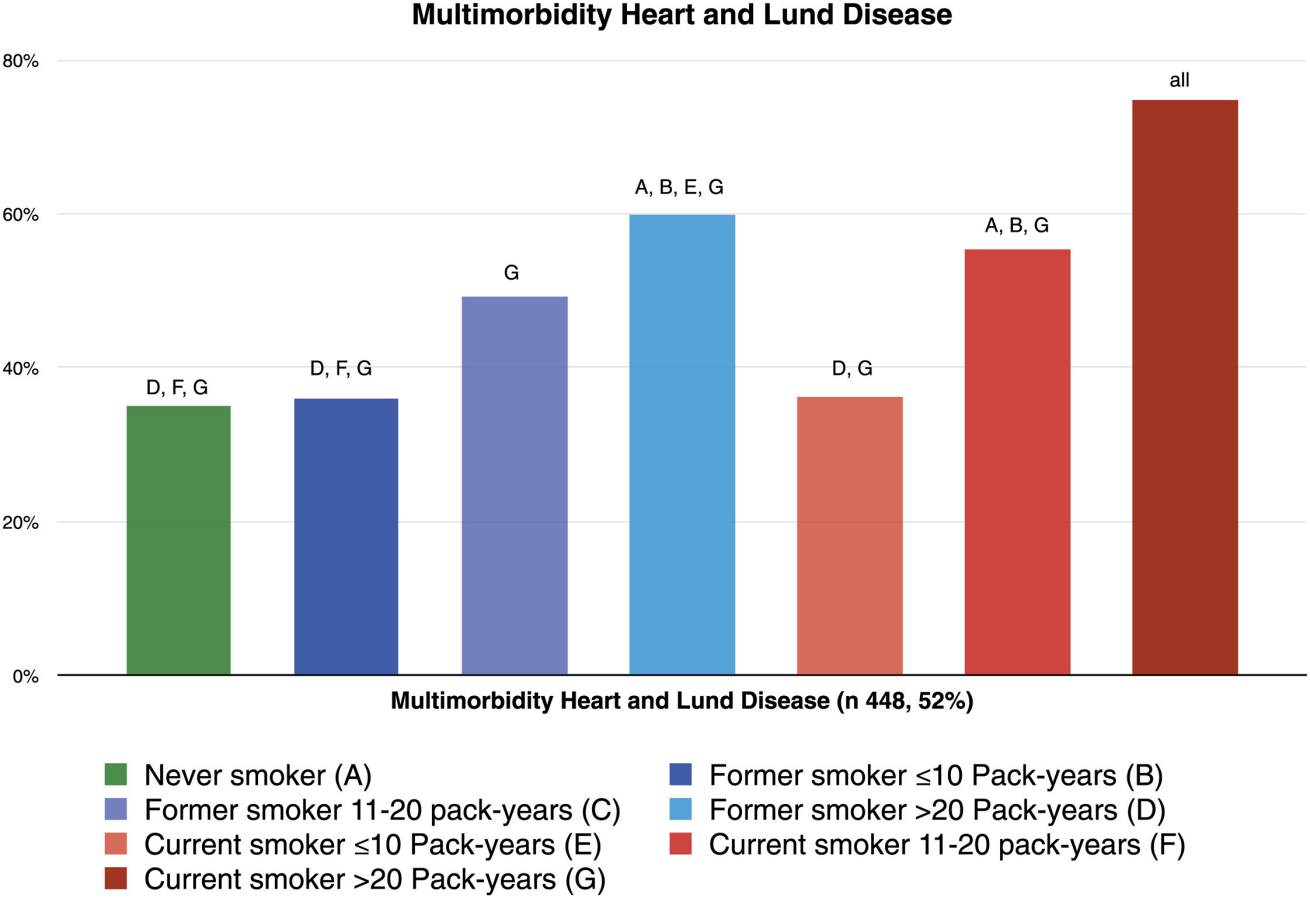
Prevalence of MLHD by smoking and pack-years groups.

**Fig 6:**
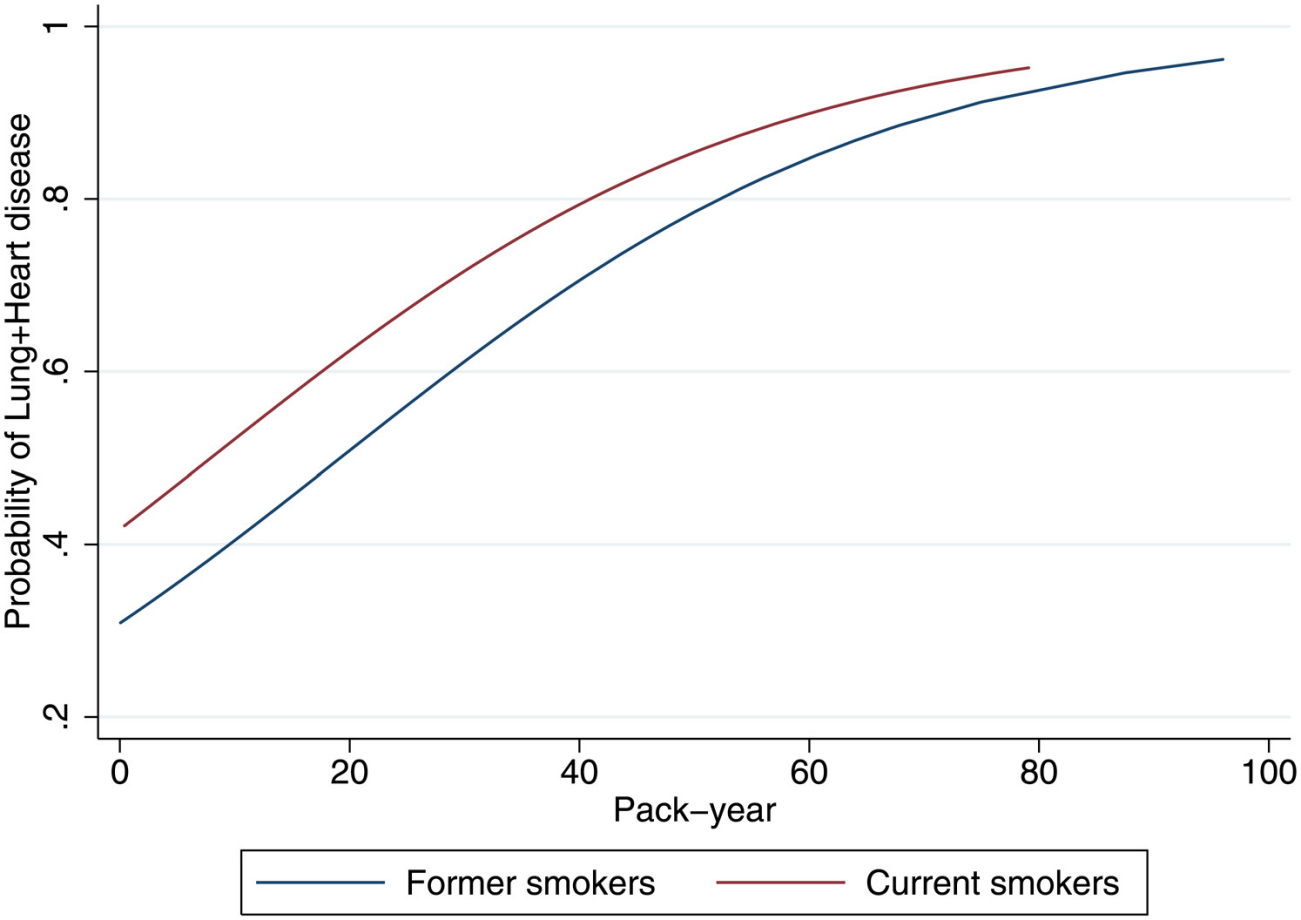
Probability of MLDH by pack-years in former and current smokers: Univariate logistic regression.

MLHD was present in 484 (53.6%) patients: 78 (36.5%) were never smokers (Fig 5). There was a trend towards a higher prevalence of MLHD with increasing pack-years of smoking, both in former (p<0.001) and in current smokers (p<0.001). The highest prevalence was observed in current smokers. Fig 6 shows the interaction of smoking status with pack-years for the risk of MLHD.

### Multivariarble and ROC analyses

**Fig 7:**
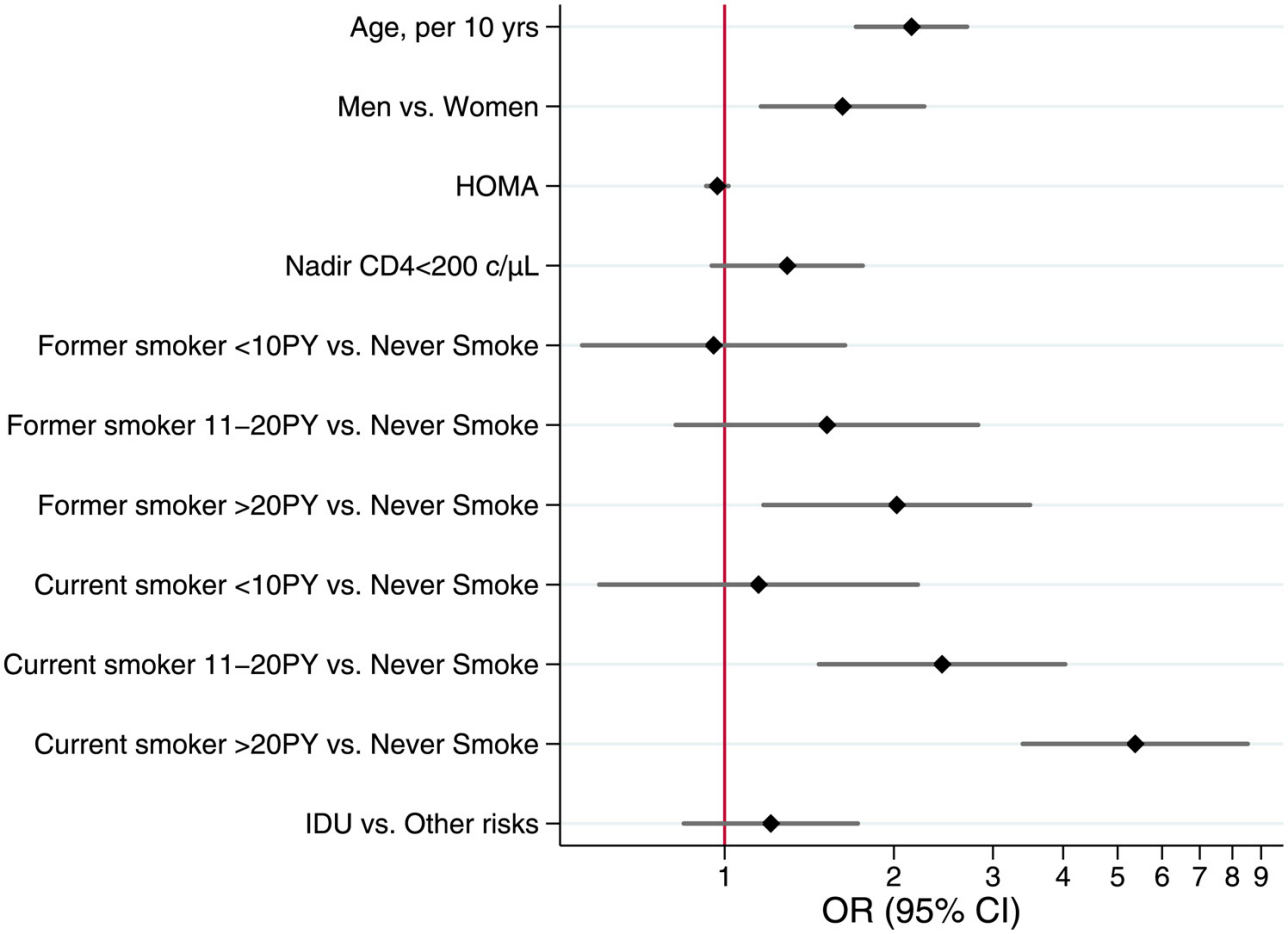
Factors associated with MLHD: Multivariable logistic regression.

A multivariable logistic regression model (Fig 7) was built to identify independent predictors of MLHD. Variables independently associated with MLHD were: age per 10-years interval increase (OR = 2.15, 95% CI:1.71–2.69), male gender (OR = 1.62, 95% CI:1.16–2.26) and FS>20PY (OR = 2.02, 95% CI:1.17–3.49), CS:11–20PY (OR = 2.44, 95% CI:1.47–4.03) and CS>20PY (OR = 5.37, 95% CI:3.39–8.51).

**Fig 8:**
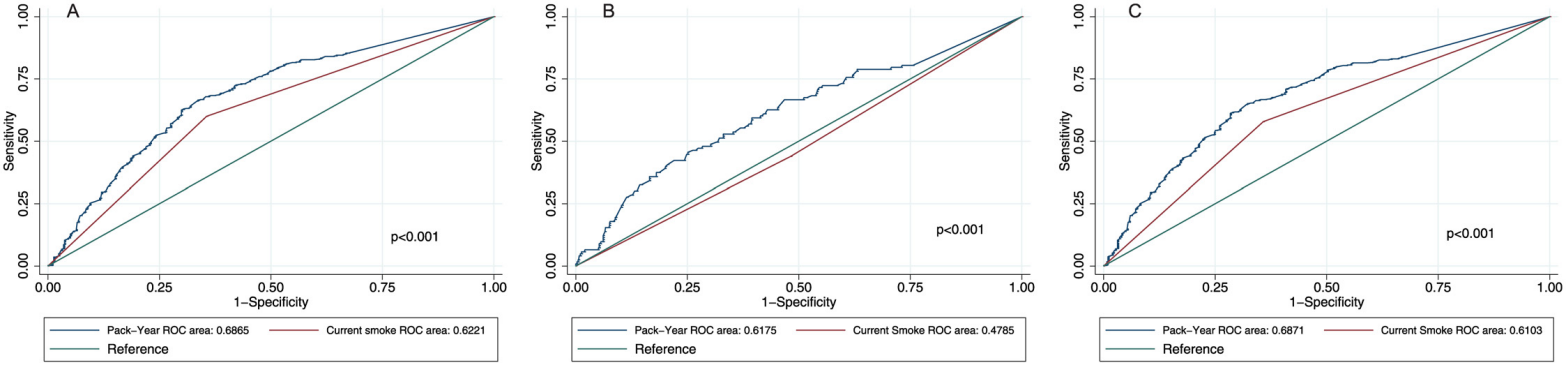
Sensitivity and specificity of pack-years and smoking status in predicting clinical and subclinical lung and heart diseases. ROC curves for sensitivity and specificity of pack-years and smoking status in clinical and subclinical lung disease (A), clinical and subclinical heart disease (B), and MHLD (C).

ROC curves were used to assess how well pack-years and smoking status predicted clinical and subclinical lung disease (Fig 8 - panel A), clinical and subclinical heart disease (Fig 8 - panel B), and MHLD (Fig 8 - panel C). Pack-years were more closely associated with clinical and subclinical MLHD than smoking status (p<0.001).

**Fig 9:**
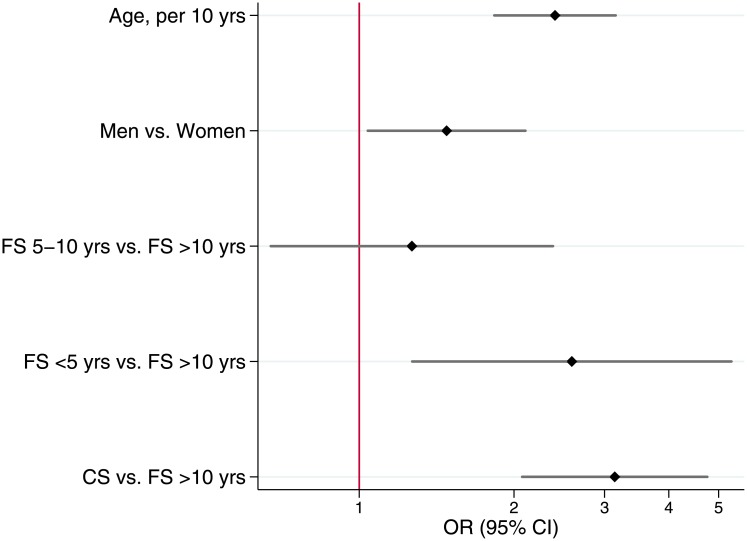
MLHD and time since smoking cessation. Multivariable logistic regression.

**Fig 10:**
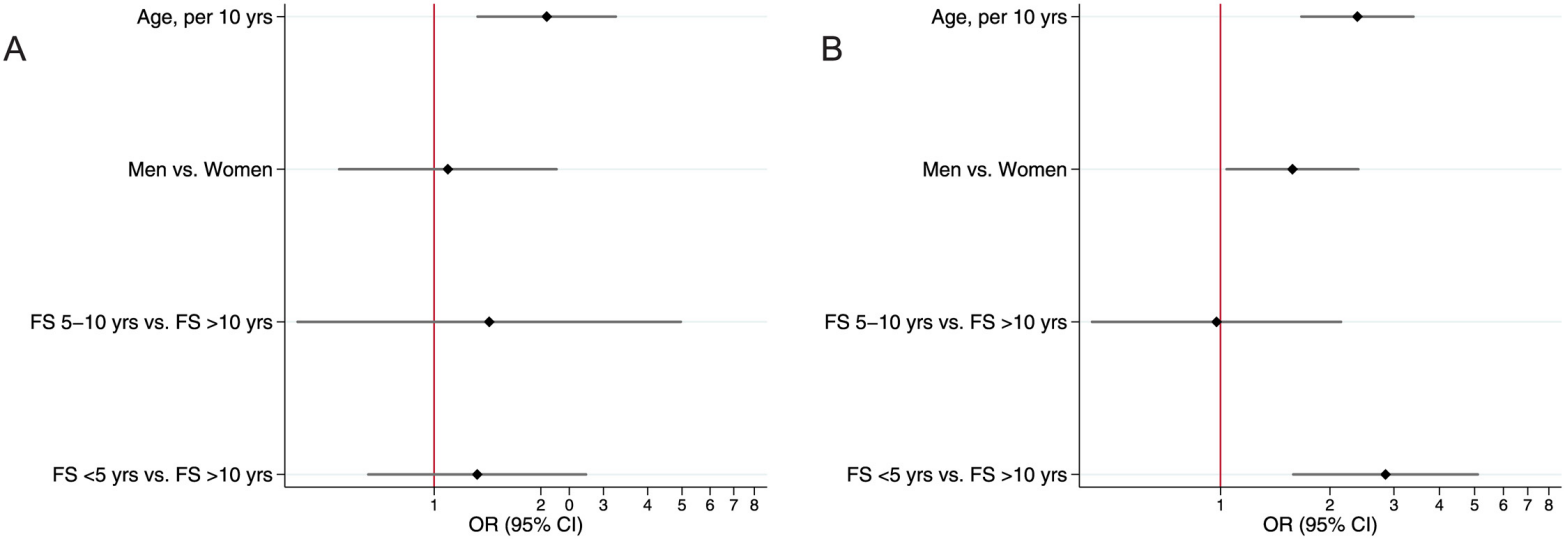
MLHD and time since smoking cessation. Multivariable logistic regression in (A) former smokers with less than 10 pack-years and (B) more than 10 pack-years smoking history.

We also evaluated whether smoking cessation modified the risk of MLHD among 259 former smokers. In this group, time since smoking cessation was <5 years in 46 (18%) patients, 5–10 years in 59 (23%) patients, and >10 years in 154 (59%) patients. In these 3 groups, the prevalence of MLHD was 61%, 46%, and 46%, respectively (p = 0.188). After adjustment for age and sex, independent predictors of MLHD were current smoking (OR 3.14, 95% CI 2.07; 4.75, p<0.001) and smoking cessation less than 5 years prior (OR 2.59, 95% CI 1.27; 5.29, p = 0.009) (Fig 9). We tested the interaction between smoking cessation and pack-years; only smoking cessation time <5 years among patients with >10 pack-years was a significant risk factor for MLHD (Fig 10).

## Discussion

In this well-characterized and prospectively followed cohort of HIV-infected patients we observed that lifetime cumulative cigarette smoking exposure was a significant and powerful predictor of clinical and subclinical cardio-pulmonary disease. We observed a high rate of current smoking and, as expected in this patient group, a very high burden of pulmonary and cardiac disease. In contrast, current smoking status performed quite poorly in predicting MLHD, suggesting that ascertaining only current smoking status, which has been the case in previous epidemiological studies, may underestimate the true adverse cardio-pulmonary impact of smoking among HIV-infected patients. Additionally, prior smoking was independently associated with a high prevalence of clinical and subclinical disease. Therefore, our data suggest that smoking pack-year history should be routinely collected in clinical and epidemiologic studies seeking to assess the health impact of tobacco use, and also highlight the importance of smoking cessation in mitigating the risk of heart and lung disease among HIV infected individuals.

Our findings corroborate those of Petoumenos et al, who showed that the risk of cardiovascular disease decreases with increasing time since smoking cessation among HIV-infected patients[22]. Our data complement and extend those findings by demonstrating that the risk of MLHD is significantly reduced after 5 years or more of smoking cessation among patients with less than 10 pack-years of smoking (Fig 10). In patients with greater cumulative cigarette exposure, the time required to achieve smoking cessation benefits may be longer.

However, these data should be interpreted with caution since we could not ascertain whether smoking cessation for some patients occurred as a consequence of medical illness as opposed to a voluntary decision. Indeed, previous studies have suggested that some smokers quit because of an acute smoking-related medical event, leading to a bias that is commonly referred to as an “ill-quitter effect”[23]. This phenomenon may spuriously elevate the apparent risk of smoking-related morbidity and mortality among former smokers during the early years following smoking cessation. There were additional limitations to our analyses; because of the observational nature of the study, there could have been a survival bias. Given that pack-year data are calculated from patient self-reporting, we cannot exclude patient bias. We also did not collect data regarding environmental tobacco smoke in non-smokers, or occupational exposure to smoke, air pollution and other cardio-pulmonary risk factors in smokers and non-smokers. Moreover, we performed pulmonary function tests in only 350 patients (38.7%) and data regarding recreational drugs was not comprehensive; our questionnaires included only information about cocaine and heroin use and not other illicit drugs such as cannabis. Finally, we did not have available for comparison a control group of HIV-uninfected patients to allow cross-comparisons.

Nevertheless, our findings are important for the following reasons: first, obtaining pack-year history of smoking is important for both former and current smokers, when assessing risk of clinical and subclinical heart and lung disease. Second, careful assessment of pack-year history of smoking may enable cost-effective screening for clinical and subclinical lung and heart disease among HIV-infected individuals. Third, smoking cessation strategies may be most effective among patients with shorter pack-year history of smoking.

## Supporting Information

S1 Dataset (XLS)

## Abbreviations

AIDS: acquired immune deficiency syndrome
ART: anti-retroviral therapy
BMI: body mass index
CAC: coronary artery calcium
COPD: chronic obstructive pulmonary disease
CT: computed tomography
CS: current smokers
CVD: cardiovascular disease
DEXA: dual energy X-ray absorptiometry
FEV_1_: forced expiratory volume in one second
FRS: Framingham risk score
FS: former smokers
FVC: forced vital capacity
HDL-C: high-density lipoprotein cholesterol
HIV: Human immunodeficiency virus
HOMA-IR: homeostasis model assessment of insulin resistance
HTN: hypertension
IQR: interquartile range
LDL-C: low-density lipoprotein cholesterol
MHMC: Modena HIV metabolic clinic
MI: myocardial infarction
MLD: multimorbidity lung disease
MLHD: multimorbidity lung and heart disease
NRTIs: nucleoside reverse transcriptase inhibitors
NNRTIs: non- nucleoside reverse transcriptase inhibitors
NS: never smokers
PIs: protease inhibitors
PY: pack-years
ROC: Receiver operating characteristics
T2DM: type 2 diabetes mellitus
